# Nurses’ Roles in Supporting Mental Health Among Preoperative Cardiac Patients in Kazakhstan: A Qualitative Study

**DOI:** 10.1155/jonm/2241221

**Published:** 2026-06-13

**Authors:** Alpamys Shaldarbek, Joseph Almazan, Ejercito Mangawa Balay-odao

**Affiliations:** ^1^ Department of Medicine, School of Medicine, Nazarbayev University, Kerey and Zhanibek Khans St 5/1, Astana, 010000, Kazakhstan, nu.edu.kz

**Keywords:** cardiovascular disease, exploratory qualitative, mental health, nurse, preoperative

## Abstract

**Introduction:**

Patients with cardiovascular disease undergoing surgery commonly experience psychological distress, including anxiety, depression, and fear of mortality, which may negatively influence surgical outcomes and recovery. However, mental health support for patients with cardiovascular disease remains unprioritized in many healthcare systems, particularly in middle‐income countries such as Kazakhstan, where mental health services are still fragmented and underdeveloped.

**Objective:**

This study aims to explore and describe the mental health promotion strategies employed by nurses when caring for preoperative patients with cardiovascular disease.

**Design:**

This study employed an exploratory–descriptive qualitative design. Data were collected through semistructured interviews with 13 nurses working in cardiac surgery units in Kazakhstan from February 3 to April 10, 2025. The interview data were analyzed using thematic analysis. The study was conducted in accordance with the Consolidated Criteria for Reporting Qualitative Research (COREQ) guidelines.

**Result:**

Initially, 96 codes were generated from the data. These codes were subsequently organized into 19 subthemes and further synthesized into four major themes: “Patient Emotional Support Needs,” “Mental Health Implementation Strategies,” “Challenges to Mental Health Promotion,” and “Influencing Factors in Mental Health Promotion.”

**Conclusion:**

Nurses play a pivotal role in promoting the mental health and psychological well‐being of patients with cardiovascular disease, with therapeutic communication and professional presence serving as fundamental components of holistic care. In Kazakhstan, this responsibility also requires adapting nursing management strategies to culturally grounded perspectives on mental health, thereby ensuring the delivery of compassionate, culturally sensitive, and comprehensive care that extends beyond physical recovery.

## 1. Introduction

Cardiovascular disease (CVD) patients undergoing cardiovascular surgical procedures frequently experience substantial psychological burden, including preoperative anxiety, depressive symptoms, and fear of mortality, which have been consistently associated with poorer perioperative outcomes and delayed postoperative recovery [1]. Such psychological distress is increasingly recognized as a clinically relevant determinant of surgical prognosis, given its association with heightened systemic inflammatory responses, increased hemodynamic instability (including elevated blood pressure), and dysregulated neuroendocrine and metabolic functioning [2].

Despite this established biopsychosocial interrelationship, the integration of structured mental health support into the preoperative management of CVD patients remains insufficiently prioritized within many healthcare systems. This challenge is particularly evident in middle‐income contexts such as Kazakhstan, where mental health services remain fragmented, inconsistently integrated into somatic care, and limited in both accessibility and institutional capacity [3]. Consequently, the absence of systematic psychological care strategies for preoperative cardiovascular patients may compromise treatment adherence, weaken engagement in lifestyle modification, and reduce participation in rehabilitation programs, thereby adversely affecting long‐term recovery outcomes. In this regard, nurses play a critical role in addressing the psychological needs of this patient population through ongoing patient interaction and the delivery of holistic care.

Notwithstanding their central role, nurses’ ability to provide effective mental health promotion and psychosocial support is frequently constrained by structural, organizational, and professional barriers. These include inadequate supportive clinical environments, insufficient institutional prioritization of mental health care, and the absence of clearly defined professional roles within multidisciplinary teams [4]. Furthermore, limited availability of human and material resources, coupled with restrictive administrative and organizational cultures, further impedes the implementation of comprehensive mental health interventions in cardiac care settings [5, 6].

In addition, deficits in specialized training in mental health care among nurses and nurse managers contribute to reduced confidence, limited competence, and inadequate preparedness to identify and address psychological distress in cardiovascular patients [5]. This educational and experiential gap may subsequently diminish motivation and engagement in mental health promotion initiatives. Moreover, inconsistencies in role delineation among nursing, medical, and psychosocial professionals may result in ambiguity regarding responsibility for psychological care, thereby fostering reluctance to initiate or sustain mental health interventions [4]. As a result, nurse managers may encounter significant challenges in coordinating multidisciplinary collaboration, ensuring role clarity, and providing effective leadership in integrating mental health services into cardiovascular care pathways.

Moreover, cultural factors, such as the stigma associated with mental health and the dominance of conventional biomedical approaches to treatment, may restrict the promotion of mental health services [7]. Cultural beliefs significantly shape perceptions of mental health, influencing patients’ willingness to disclose psychological distress, seek professional help, and accept nonsomatic forms of intervention. In many contexts, mental health problems are still viewed through a lens of personal weakness or social undesirability, resulting in avoidance behaviors and delayed help‐seeking [8].

In addition, insufficient cultural competence among healthcare professionals may further exacerbate these challenges by contributing to misinterpretation of psychological symptoms, lack of recognition of emotional distress, and the implementation of interventions that are not fully aligned with patients’ sociocultural values and beliefs [9]. Consequently, the absence of culturally responsive care may reduce the effectiveness of mental health interventions and limit patient engagement in psychosocial support strategies.

Therefore, integrating cultural competence into nursing education, clinical practice, and health policy is essential to ensure equitable, acceptable, and effective mental healthcare delivery. In Kazakhstan, these challenges are further compounded by systemic limitations, including inadequate exposure to psychological and psychiatric training within nursing and medical curricula, limited consultation time that impedes comprehensive psychosocial assessment, and insufficient interdisciplinary collaboration among nursing, medical, and mental health professionals [3]. Collectively, these factors reinforce the ongoing marginalization of psychological care within cardiovascular treatment pathways and highlight the need for more integrated, culturally sensitive, and system‐level interventions.

These findings highlight the multifactorial nature of challenges associated with integrating mental health care into preoperative cardiovascular settings. These constraints do not operate in isolation but interact across system, provider, and patient levels, ultimately limiting the consistent delivery of psychological support to CVD patients undergoing surgery.

In this context, the present study is conceptually grounded in the biopsychosocial model and stress and coping theory, which together offer a comprehensive framework for understanding the dynamic interplay between biological, psychological, and social determinants of health outcomes [10, 11]. The biopsychosocial model underscores that the preoperative experience of patients with CVD extends beyond physiological pathology and is significantly influenced by emotional states, cognitive appraisals, interpersonal relationships, and broader sociocultural environments [10]. This perspective aligns with the previously identified barriers, particularly those related to organizational structures, professional roles, and cultural beliefs that shape both care delivery and patient help‐seeking behaviors.

Complementarily, stress and coping theory provides a more process‐oriented explanation of how individuals evaluate and respond to surgical stressors [11]. It posits that patients’ cognitive appraisal of the impending surgical procedure, combined with their available coping resources, determines their psychological adjustment and subsequent recovery outcomes [11]. In the preoperative context, ineffective coping may intensify anxiety and fear, whereas adaptive coping strategies can enhance resilience and improve postoperative recovery trajectories.

These theoretical foundations position nurses as key facilitators in bridging the gap between psychological need and the delivery of clinical care. Their role extends to the systematic assessment of patients’ emotional responses, the promotion of adaptive coping mechanisms, and the mitigation of social and cultural barriers that may hinder psychological well‐being. Importantly, integrating these theoretical perspectives into nursing practice supports a shift toward more holistic, patient‐centered cardiovascular care, where mental health is recognized as an essential component of surgical preparation and recovery rather than a secondary consideration.

Despite nurses’ essential role in mental health interventions, this area remains underexplored in Kazakhstan. Existing literature lacks a comprehensive investigation of how nurses integrate mental health strategies into clinical practice, the challenges they encounter, and the specific approaches they employ. Furthermore, the healthcare system in Kazakhstan has undergone significant development since independence; however, mental health service delivery remains relatively underdeveloped [12]. The country continues to experience a high prevalence of CVD alongside limited integration of mental health services within cardiac care [13]. However, it was noted that mental health resources are concentrated primarily in urban areas, resulting in limited accessibility for rural populations [14]. Nursing education in Kazakhstan also lacks standardized mental health training, leaving many nurses insufficiently prepared to address the psychological needs of patients [3]. Therefore, exploring and describing how Kazakh nurses promote mental health among preoperative cardiovascular patients is essential to understand how healthcare policies, resource limitations, and cultural attitudes influence the integration of mental health promotion within the country’s cardiovascular care framework.

### 1.1. Aim of the Study

This study aims to explore and describe the mental health promotion strategies employed by nurses when caring for preoperative patients with CVD.

## 2. Methods

### 2.1. Design

This study employed an exploratory–descriptive qualitative design and followed the Consolidated Criteria for Reporting Qualitative Research (COREQ) guidelines to ensure rigorous reporting.

### 2.2. Setting and Participants

This study was conducted in a tertiary hospital in Kazakhstan with a 200‐bed capacity. The hospital comprises six surgical units and two intensive care units, each with an 18‐bed capacity, and is equipped with continuous life‐support monitoring. Data were collected in cardiac surgery units, selected for their role in providing care and preparing patients physically and psychologically for surgical interventions.

The study was conducted in a single hospital to ensure contextual depth and consistency in exploring participants’ experiences. Focusing on a single institution enabled a more nuanced understanding of shared practices, organizational culture, and clinical dynamics that influence the phenomenon under investigation. This approach is consistent with qualitative inquiry, where depth of insight is prioritized over breadth and transferability [15].

To enhance the depth and breadth of the data, maximum variation sampling was used to recruit nurses with diverse characteristics, including gender, unit assignment, years of experience, and highest educational attainment. This approach enabled the capture of a wide range of perspectives and minimized the risk of a homogenous sample.

The sample size was determined by data saturation. Saturation was reached after the 12^th^ interview, when no new themes, codes, or meaningful insights emerged. One additional interview (total *n* = 13) was conducted to confirm the consistency and robustness of the identified themes.

### 2.3. Data Collection

Data collection was conducted from February 3 to April 10, 2025, using semistructured, face‐to‐face interviews. Following ethical approval, a staged recruitment process was implemented within the hospital setting to ensure systematic and orderly participant enrollment. Formal permission was first obtained from hospital administrators, nurse supervisors, and head nurses. Eligible participants were identified according to predefined inclusion criteria, and head nurses initially approached potential participants to introduce the study. The researchers then contacted nurses who expressed interest and arranged face‐to‐face meetings to provide comprehensive information about the study and to obtain written informed consent. Interviews were scheduled at a convenient time for the participants to minimize disruption to clinical duties.

The interview guide was developed based on a comprehensive review of relevant literature and aligned with the study objectives. It was subsequently reviewed for face validity by experts in nursing research and mental health to ensure clarity, comprehensibility, and relevance to the intended research aims. The guide questions were then pretested with three nurses who met the inclusion criteria but were not included in the final sample. The pretest aimed to assess the clarity, flow, and comprehensibility of the questions, and minor revisions were made accordingly. Following these refinements, the finalized interview guide was used to collect data. Data obtained during the pretest were not included in the final analysis.

All interviews were conducted by a single trained researcher (AS), who had prior experience in qualitative interviewing. Before each interview, participants were informed about the study’s purpose, assured of confidentiality and anonymity, and reminded of their right to withdraw at any time without any consequences. Written informed consent was obtained for audio recording and for the use of selected verbatim quotations in the manuscript and subsequent publication before data collection. No repeat interviews were conducted.

Interviews were conducted in a quiet, private consultation room within the hospital, with only the researcher and participant present to ensure confidentiality and minimize interruptions. Each interview lasted approximately 30–60 min and was audio‐recorded with participants’ consent. Field notes were documented immediately after each interview to capture nonverbal cues, contextual details, and preliminary analytical reflections.

All interviews were conducted in Russian to enable participants to express their experiences comfortably in their native language. The audio recordings were transcribed verbatim in Russian. To prepare the data for analysis and reporting in English, a forward–backward translation procedure was undertaken. Initially, the Russian transcripts were translated into English by a bilingual expert (forward translation). Subsequently, a second independent bilingual translator translated the English version back into Russian (back translation) to ensure accuracy and conceptual equivalence. Discrepancies between the original and back‐translated versions were systematically examined and discussed by the research team. Minor inconsistencies, particularly in idiomatic expressions and technical nursing terminology, were resolved by consensus to preserve the meaning and contextual nuance of participants’ accounts.

Data collection and preliminary analysis were conducted concurrently and continued until data saturation was achieved, at which point no new relevant information or themes emerged.

### 2.4. Data Analysis

Braun and Clarke’s [16] six‐phase thematic analysis framework was employed to systematically identify, analyze, and interpret patterns within the data. This approach offered a flexible yet rigorous framework for describing nurses’ experiences in promoting mental health among cardiovascular patients.

In the familiarization phase, all three researchers independently read and reread the transcript file multiple times to achieve immersion in the data and document initial impressions. During the generation of initial codes, an inductive, data‐driven approach was adopted, allowing codes to emerge directly from the data rather than being pre‐established. Each transcript was independently coded line‐by‐line by the three researchers, with meaningful units of text assigned initial codes aligned with the study objectives. The coding framework was developed iteratively and continuously refined throughout the analysis process.

During the theme‐search phase, the researchers collaboratively examined and clustered codes into potential themes based on patterns and conceptual similarities across the dataset. These themes were subsequently reviewed and refined through iterative discussions among the three researchers to ensure coherence, consistency, and alignment with both coded extracts and the full dataset. Any discrepancies in coding or theme development were initially discussed to reach consensus; when disagreements persisted, code definitions were revisited, and the data were re‐examined until agreement was achieved.

In the defining and naming phase, themes were further refined to clearly delineate their scope and essence, ensuring that each theme was distinct, conceptually coherent, and representative of the data. All three researchers agreed upon final themes. In the final phase, representative quotations were selected, and a coherent analytical narrative was developed to present the findings, ensuring that the interpretation accurately reflected participants’ experiences and remained aligned with the study objectives.

### 2.5. Ethical Consideration

This study was approved by the Institutional Research Ethics Committee (IREC) of Nazarbayev University School of Medicine (2024Nov#01). Written consent was obtained from all participants after verbalization of their understanding of the study’s purpose, its voluntary nature, and their right to withdraw at any time without repercussions.

Confidentiality was maintained at all times. Personal data were anonymized, and alphanumeric identifiers were used when reporting the study results. The data collected from the interviews were securely stored on the primary investigator’s password‐protected personal computer and will be retained for 3 years. The Declaration of Helsinki was observed during the research process.

### 2.6. Rigor and Trustworthiness

In the study, several strategies were implemented to ensure rigor and trustworthiness. First, credibility was established through prolonged engagement with nurses, enabling a deep understanding of their experiences. Second, transferability was ensured by providing thick descriptions of the context and participants, enabling others to assess the applicability of the findings to different settings. Dependability was addressed by maintaining a clear record of the research process, including decision‐making steps, changes to the research design, and data collection and analysis procedures. Finally, confirmability was strengthened by practicing reflexivity, documenting personal biases, and presenting findings to participants to verify the accuracy of the analysis.

## 3. Results

The study participants comprised 13 nurses working in the cardiac surgical unit. The sample included nine female and four male nurses. Participants’ clinical experience in caring for patients with CVD ranged from 1 to 5 years. All participants held a bachelor’s degree (see Table 1).

**TABLE 1:** Demographic profile of the participants.

Participant	Gender	Unit assignment	Years of experience working with CVD patients	Highest educational attainment
Nurse 1	Female	^∗^CSU	2	Bachelor
Nurse 2	Female	CSU	2	Bachelor
Nurse 3	Male	CSU	3	Bachelor
Nurse 4	Female	CSU	7	Bachelor
Nurse 5	Male	CSU	5	Bachelor
Nurse 6	Male	CSU	1	Bachelor
Nurse 7	Female	CSU	2	Bachelor
Nurse 8	Female	CSU	2	Bachelor
Nurse 9	Female	CSU	1	Bachelor
Nurse 10	Female	CSU	5	Bachelor
Nurse 11	Male	CSU	3	Bachelor
Nurse 12	Female	CSU	6	Bachelor
Nurse 13	Female	CSU	5	Bachelor

^∗^Cardiac Surgery Unit.

**FIGURE 1:**
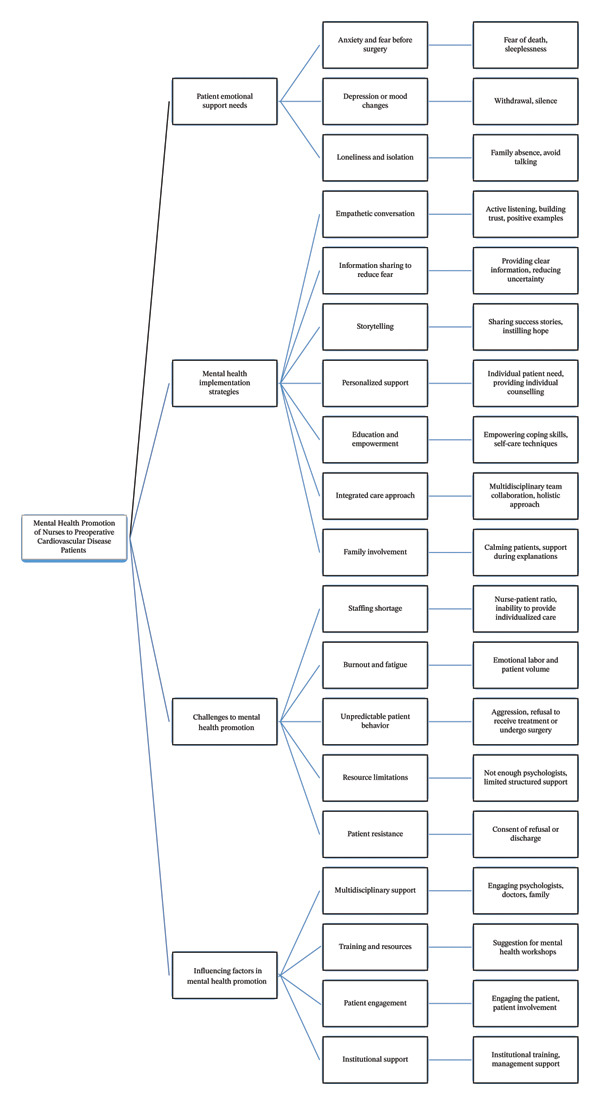
Summary of themes, subthemes, and codes.

This study explored and described nurses’ mental health promotion practices in caring for preoperative patients with CVD. The initial phase of analysis generated 96 codes, which were subsequently synthesized and organized into 19 subthemes. These subthemes were further clustered into four overarching themes. The final themes identified were as follows: “Patient Emotional Support Needs,” “Mental Health Implementation Strategies,” “Challenges to Mental Health Promotion,” and “Influencing Factors in Mental Health Promotion” (see Figure 1; Table 2).

**TABLE 2:** Summary of subthemes and themes.

Subthemes	Themes
Anxiety and fear before surgery	Patient emotional support needs
Mood changes	
Loneliness and isolation	

Empathetic conversation	Mental health implementation strategies
Information sharing to reduce fear	
Storytelling	
Personalized support	
Education and empowerment	
Integrated care approach	
Family involvement	

Staffing shortage	Challenges to mental health promotion
Burnout and fatigue	
Unpredictable patient behavior	
Resource limitations	
Patient resistance	

Multidisciplinary support	Influencing factors in mental health promotion
Training and resources	
Patient engagement	
Institutional support	

### 3.1. Theme 1: Patient Emotional Support Needs

This theme reflects the psychosocial burden experienced by patients undergoing cardiovascular surgery. The emotional trajectory spans from anticipatory anxiety to postoperative adjustment struggles. Consequently, preoperative patients represent a particularly vulnerable group within hospital settings; however, their psychological needs are often insufficiently addressed in routine clinical care.

### 3.2. Anxiety and Fear Before Surgery

Many nurses reported that patients frequently experienced and verbalized anxiety, even during hospitalization, as well as fear of surgery and concerns regarding postoperative survival. In some cases, patients were unable to sleep due to heightened fear, which contributed to significant emotional distress. Nurses addressed these concerns by anticipating patients’ questions and providing information that the medical and surgical team will be monitoring their conditions.

“Many patients are often afraid of surgery or diagnosis. They often ask questions about their condition and the surgical procedure to be done, and are anxious about possible side effects.” Nurse 1

### 3.3. Mood Changes

Preoperative patients were identified as a particularly vulnerable group, with some expressing profound feelings of helplessness and consequent fear regarding the surgical procedure. Nurses further reported observable indicators of depressive states, including withdrawal and, in some cases, agitation. These emotional changes were not always explicitly verbalized by patients; however, they were evident through behavioral cues during inpatient care.

“When they woke up, they were very agitated… Some showed deep feelings of helplessness and fear about surgery, and some have a low mood and are irritable. These emotional changes are not always verbalized, but we can clearly see them in their behavior during hospitalization.” Nurse 8

### 3.4. Loneliness and Isolation

Cardiac patients undergoing surgical procedures often experience prolonged preoperative hospitalization, which can result in increased emotional and social detachment from their families. As hospital stay duration increases, patients frequently report diminished perceived emotional support. This sense of isolation is further exacerbated by the absence of regular visitors and limited privacy, particularly in shared ward settings. Nurses observed that being alone in the hospital environment contributed to heightened anxiety and sadness among several patients.

“Patients often feel isolated after surgery, especially when family members are not allowed to visit.” Nurse 10

### 3.5. Theme 2: Mental Health Implementation Strategies

Nurses employ a range of strategies to promote mental health among cardiovascular patients, with communication identified as a central intervention. In particular, empathetic communication was highlighted as essential in reducing patient anxiety, fostering trust, and facilitating understanding through clear explanations of procedures.

### 3.6. Empathetic Conversation

Nurses utilized verbal reassurance and active listening as key strategies to alleviate patient anxiety and provide emotional support. Nurses identified the establishment of trust as a fundamental component of effective nurse–patient communication.

“Even a 5‐minute conversation helps. I explain their condition and what to expect. Once they understand it’s manageable, they relax. I noticed that building trust is very important in communicating with patients.” Nurse 5

### 3.7. Information Sharing to Reduce Fear

Nurses emphasized that reducing anxiety requires clear, structured, and honest explanations of the procedure. They further noted that overly generalized reassurance may undermine patient trust; instead, transparent and accurate communication was considered more effective in fostering understanding and psychological comfort.

“I explain their condition, what to expect. Once they understand it’s manageable, they relax.” Nurse 11

### 3.8. Storytelling

Nurses used storytelling by sharing patients′ experiences of similar surgical procedures to instill hope and alleviate fear. They observed that presenting positive examples and successful surgical outcomes fostered optimism among patients, which, in turn, contributed to improved emotional well‐being and was perceived to support recovery.

“We primarily use communication skills — we talk to them, explain the nature of their disease just like sharing a story, and we share stories of those patients with successful surgical procedures.” Nurse 9

### 3.9. Personalized Support

Nurses observed that personalized support was essential in promoting patients’ mental health. Tailoring interventions to each patient’s specific needs and circumstances, such as providing counseling on stress management and facilitating access to mental health resources, was considered particularly effective in addressing psychological distress.

“Patients have different needs, and we make sure that we provide care based on the needs of the patient.” Nurse 12

### 3.10. Education and Empowerment

Educating patients about the relationship between mental health and cardiovascular conditions, as well as equipping them with coping strategies and self‐care techniques, was identified as an effective approach to promoting patients’ mental well‐being. Nurses further emphasized that structured health education is essential to ensure patients fully understand the planned procedure, which in turn helps to alleviate anxiety and enhance psychological preparedness.

“Sometimes a patient asks the same question multiple times, so it is important to educate the patient on the psychological effects of their condition and intervention, to decrease their anxiety.” Nurse 1

### 3.11. Integrated Care Approach

An integrated care approach was identified as essential for addressing patients’ psychological needs, which often extend beyond the scope of routine care provided by individual healthcare professionals. Nurses emphasized that collaboration within multidisciplinary teams, including psychologists and social workers, is crucial in ensuring a holistic approach to patient care that adequately addresses both physical and psychological dimensions of health.

“I engaged a doctor or psychologist so they could explain everything to the patient.” Nurse 9

### 3.12. Family Involvement

Family involvement was identified as crucial in supporting and sustaining patients’ mental health. Nurses observed that family members often served as a primary source of emotional support, contributing to reduced anxiety and the development of adaptive coping strategies. In particular, patients frequently relied on their families for comfort, especially in situations where the absence of loved ones intensified feelings of distress and vulnerability.

“Some patients calm down after a video call with their family. It’s a simple yet effective way of improving their mood.” Nurse 3

### 3.13. Theme 3: Challenges to Mental Health Promotion

Nurses identified several issues that slowed effective mental health promotion. The nurse–patient ratio, heavy workloads, and the absence of structured mental health support were consistently identified as key barriers to effective mental health promotion.

### 3.14. Staffing Shortage

Nurses reported that staffing shortages limited the time spent with each patient, as they were required to attend to multiple patients simultaneously, thereby reducing their capacity to provide adequate emotional support and engage in effective mental health promotion.

“When I have 8 or 9 patients, I physically can’t give everyone the emotional support they need.” Nurse 10

### 3.15. Burnout and Fatigue

Nurses identified emotional labor, night shifts, and exposure to patient deaths as primary sources of fatigue and emotional disengagement. Some nurses described feeling emotionally “drained,” which negatively affected their empathy and ability to communicate effectively with patients. In addition, heavy workloads limited nurses’ capacity to adequately address patients’ emotional needs, which was reported to contribute to patient dissatisfaction.

“We face burnout too, and it’s hard to support others if you’re emotionally drained.” Nurse 4

### 3.16. Unpredictable Patient Behavior

Unpredictable patient behavior was identified as a barrier to providing effective emotional support. Several nurses reported that some patients were not receptive to emotional interventions, with instances of resistance to support, refusal of medication, and occasional aggressive behavior.

“If a patient is unusually quiet, avoids eye contact, or doesn’t want to eat, and refuses to talk to us.” Nurse 3

### 3.17. Resource Limitations

Nurses mentioned that insufficient resources, such as limited access to mental health professionals and educational materials, hinder the effectiveness of mental health promotion.

“We have a limited number of psychologists to support the patients’ needs, so their psychological needs are not fully addressed.” Nurse 13

### 3.18. Patient Resistance

Patient resistance was identified as a barrier to mental health interventions. Nurses reported that this resistance was often associated with stigma, limited awareness, and personal beliefs, as well as fear, reluctance to discuss emotional needs, and misinformation. Consequently, some patients declined or were unwilling to engage in necessary care.

“Some patients refuse treatment and sign a refusal for treatment form due to misinformation.” Nurse 9

### 3.19. Theme 4: Influencing Factors in Mental Health Promotion

Several factors influence the effectiveness of mental health promotion strategies. Nurses often emphasize that collaboration with physicians and psychologists is a crucial component of patient care, addressing both physical and psychological dimensions of treatment.

### 3.20. Multidisciplinary Support

The participants noted that the most effective care environment is characterized by coordinated efforts among nurses, physicians, psychologists, and other members of the healthcare team. This collaborative approach was noted to be effective in managing high‐risk or emotionally distressed patients. Many nurses reported that sharing responsibilities with other healthcare professionals is needed to ensure continuity of care and support patients’ mental health needs.

“Some patients were very anxious about their situation and not open to communication with me, and in that situation, I engaged a doctor or psychologist so they could explain everything to the patient.” Nurse 11

### 3.21. Training and Resources

Nurses identified mental health knowledge and training as key factors influencing their ability to implement effective strategies. Thus, they noted that to strengthen their capacity to deliver effective mental health care, hospital and nurse leaders should provide access to adequate resources and opportunities for continuous professional development.

“To improve the emotional care of the patient, the hospital should organize mental health workshops for nurses.” Nurse 4

### 3.22. Patient Engagement

Nurses identified patient engagement as a factor influencing the success of mental health promotion interventions. Patients’ willingness to participate in such activities and their receptivity to counseling were considered crucial in determining the effectiveness of these interventions.

“To effectively provide care, we try to engage patients and provide support during those times.” Nurse 7

### 3.23. Institutional Support

Healthcare institutions’ policies and organizational support, including the allocation of time for mental health‐related activities and the availability of mental health specialists, were considered pivotal in either facilitating or hindering nurses’ efforts.

“The support from the hospital is needed, for example, conducting training and seminars to enhance nurses’ skills in mental health.” Nurse 7

## 4. Discussion

The study shows the nurses’ mental health promotion practices for preoperative CVD patients. The findings highlight several key themes that reflect the strengths and challenges within the current healthcare system.

The findings show that nurses observed preoperative CVD patients need emotional support due to anxiety, fear, and emotional vulnerability. The significant emotional distress reported among preoperative CVD patients is attributed to systemic issues, such as limited mental health services and prolonged hospital stays, which isolate patients from familial support [3]. The prolonged hospital stays and restricted visitation policies hinder familial support, increasing patients′ feelings of isolation. According to Wan et al. [17], family has long been seen as an important factor against psychological distress, and its absence during the preoperative phase can diminish the emotional strength and recuperative capacity of patients. Thus, the convergence of psychological vulnerability, systemic deficiencies in service delivery, and environmental marginalization exacerbates patient vulnerability, thereby substantially compromising overall well‐being [18, 19]. These observations can be understood through the biopsychosocial model, which emphasizes how biological (CVD and surgical stress), psychological (anxiety and fear), and social (family support, hospital environment) factors interact to affect patients’ mental health [10]. Stress and coping theory also helps explain how these stressors increase patients’ perceived stress and the need for effective coping strategies [11]. This finding suggests that nurse managers should ensure that nurses are adequately equipped, motivated, and supported to deliver effective mental health interventions.

Nurses use different approaches to support the mental well‐being of CVD patients in the preoperative period. Among these, empathic communication is identified as one of the most effective strategies, encompassing active listening, open‐ended dialog, and tailored information sharing, all grounded in a therapeutic nurse–patient relationship and contributing to trust‐building. Borkowski and Borkowska [20] emphasize that empathic communication strategies enhance patient confidence, alleviate fear, and address emotional needs, thereby playing a central role in psychological support. In addition, sharing narratives of successful surgical recoveries was reported to instill hope and provide emotional comfort during the preoperative phase [21]. This individualized and emotionally responsive approach strengthens patient education and promotes psychological well‐being. From the perspective of stress and coping theory, these interventions enhance patients’ coping resources, reduce perceived stress, and facilitate adaptive emotional regulation during the preoperative period [11]. Overall, these findings underscore the importance of equipping nurses with advanced communication skills, psychological assessment competencies, and effective emotional support strategies.

Despite nurses’ best efforts, they encounter significant barriers in providing effective mental health support. Staffing shortages and high patient‐to‐nurse ratios limit the time available for emotional care, resulting in unequal attention to patients and contributing to patient dissatisfaction. Inadequate staffing has been consistently identified as a primary occupational stressor among mental health nurses, directly compromising their capacity to deliver high‐quality care [22, 23]. In addition, the unpredictable nature of patient behavior, including resistance to treatment and episodes of aggression, further complicates the delivery of psychological support. This finding is consistent with Kemp et al. [24], who reported that nurses frequently encounter patient aggression, treatment refusal, and emotional distress, all of which hinder the provision of effective psychological care.

Moreover, nurses highlighted resource constraints, particularly limited access to mental health professionals and educational materials, as additional factors impeding effective intervention. These challenges may be interpreted through the biopsychosocial model, which underscores the interaction between systemic (social), psychological, and individual factors in shaping care delivery [10]. Concurrently, stress and coping theory elucidates the additional burden of occupational stress experienced by nurses, which may adversely affect the quality and consistency of care provided [11]. Collectively, these challenges reflect broader systemic limitations within Kazakhstan’s healthcare infrastructure [3].

Another important finding was the significant role of family members in facilitating the mental health of preoperative CVD patients. Family involvement in mental health promotion was found to be essential, as it contributes to emotional regulation, shared decision‐making, and treatment adherence. Almazan et al. [25] similarly reported that family involvement reduces anxiety and enhances patients’ sense of safety. However, it was also noted that, in Kazakhstan, family members are permitted to visit their hospitalized relatives during designated visiting hours [26], which provides structured opportunities for emotional support and reassurance; however, this arrangement may limit continuous family presence during periods of heightened patient psychological vulnerability. Thus, this finding underscores the importance of family‐centered care in improving patient outcomes, as family members play a crucial role in supporting patients’ emotional adjustment and recovery following surgical procedures. The biopsychosocial model supports this notion, as family support represents a key social determinant that buffers stress and promotes psychological well‐being [10]. Similarly, stress and coping theory further explains how family involvement strengthens patients’ adaptive coping mechanisms during stressful situations, such as the preoperative period [11]. Collectively, these findings suggest that nurse leaders should consider developing policies that allow at least one family member to remain with patients beyond standard visiting hours to enhance emotional support further and improve care outcomes.

Nurses also observed that when patients were confused about their diagnosis or treatment, or experienced heightened tension, they were less likely to make decisions independently and instead deferred to their family members or sought reassurance from them. In this context, family members functioned as interpreters, advocates, and co‐decision‐makers, assisting patients in understanding and coming to terms with their health condition. Corrigan and Lee [27] similarly noted that increased family involvement in patient education enhances patients’ understanding of illness, thereby improving shared decision‐making and coping, particularly in culturally familial care settings. This finding also aligns with the biopsychosocial model, which emphasizes the role of social determinants in shaping health outcomes, as well as with stress and coping theory, which highlights how support systems, such as family involvement, strengthen patients’ capacity to manage stress and emotional challenges [10, 11].

Nurses identified several factors influencing the effectiveness of mental health promotion strategies. Multidisciplinary collaboration among nurses, physicians, and psychologists was recognized as essential for delivering comprehensive patient care. Shared responsibility among healthcare professionals facilitates a holistic approach to addressing patients’ psychological needs. Patients with CVDs are more likely to experience comorbid psychiatric conditions, particularly anxiety and depression, which are highly prevalent in perioperative settings [28]. Nie et al. [29] similarly emphasize that comprehensive care involving physicians, psychologists, and social workers promotes coordinated and patient‐centered management. The integration of diverse professional expertise helps mitigate emotional burden among nurses while enhancing the effectiveness of interventions [30, 31]. From a theoretical perspective, multidisciplinary collaboration supports a holistic approach to care by addressing the biological, psychological, and social dimensions outlined in the biopsychosocial model, with each professional contributing specialized expertise to meet patients’ complex needs [10]. Such collaboration also provides nurses with essential support in managing complex and emotionally demanding clinical situations. Accordingly, nurse managers should ensure that multidisciplinary collaboration among nurses, psychologists, physicians, and families is properly coordinated to optimize preoperative support and patient outcomes. This underscores the importance of managerial leadership in facilitating structured communication and teamwork within healthcare settings.

Training and access to resources were also identified as critical determinants of nurses’ ability to implement mental health interventions effectively. Nurses’ capacity to promote mental health is closely linked to their knowledge, confidence, and access to appropriate tools [32]. Many nurses reported feeling insufficiently prepared due to a lack of formal training in psychological support, resulting in reliance on specialists to address patients’ emotional needs. Within the biopsychosocial model, such training strengthens nurses’ ability to address both psychological and social dimensions of care [10]. Similarly, stress and coping theory suggests that enhanced knowledge and skills reduce occupational stress and improve coping when managing emotionally vulnerable patients [11]. This finding highlights the responsibility of nursing management to establish and enforce standards of care that integrate physical and mental health components.

Furthermore, patient engagement and institutional support, particularly through policies that promote mental health activities, were identified as essential in fostering a therapeutic care environment. These findings align with global health recommendations advocating for integrated care models and continuous professional development to improve patient outcomes [33, 34]. Nurses emphasized that mental health interventions are more effective when patients are actively engaged, well‐informed, and supported by both healthcare professionals and family members. Integrating these findings within the biopsychosocial model highlights the importance of addressing biological, psychological, and social dimensions of care [10, 35]. Similarly, stress and coping theory underscores how patient engagement and supportive systems enhance coping capacity and resilience during the preoperative period [11]. Therefore, this study suggests implementing structured mental health screening, counseling services, and emotional support protocols as part of quality improvement initiatives in cardiac care units.

### 4.1. Limitations of the Study

When interpreting these results, it is important to note several limitations of this study. First, the study was conducted in a specialized medical institution in Astana, which limits the transferability of the findings to other healthcare settings, particularly those with different organizational structures, levels of resources, or care delivery systems. Second, given that it is a qualitative study, the results are based on the subjective experiences and perceptions of the nurse participants. Third, the study is limited to exploring the perceptions of only one category of healthcare professionals, i.e., “nurses,” without perspectives from other relevant stakeholders (e.g., patients, doctors, psychologists, and so on), which may have enriched the comprehensiveness of the analysis. Finally, the study does not account for how institutional policies, cultural norms, or patient demographics affect nurses’ mental health practices.

## 5. Implications for Nursing Management

The findings of this study highlight the central role of nursing leadership in enhancing psychological care for patients awaiting cardiac surgery. Integrating mental health support into preoperative pathways requires strong governance, strategic coordination, workforce development, and supportive organizational frameworks.

First, nurse managers should prioritize ongoing professional education focused on psychological care, therapeutic communication, anxiety management, and emotional support strategies for cardiac patients. Such initiatives are essential to strengthen nurses’ competence and confidence in identifying psychological distress, including fear, anxiety, and depressive symptoms commonly experienced before surgery. In this regard, workshops and simulation‐based learning can further enhance clinical preparedness to deliver holistic care.

Second, standardized mental health screening procedures should be incorporated into routine preoperative assessment. The use of validated instruments for detecting anxiety, depression, and stress would support early recognition of at‐risk patients. Embedding these tools within documentation systems and preoperative checklists may improve consistency in psychosocial assessment and facilitate timely intervention.

Third, clearly defined referral systems to psychologists, psychiatrists, and counselors should be formally established within cardiac services. Strengthening interprofessional collaboration is essential to ensure that patients with significant emotional distress receive timely specialist input and follow‐up. Structured referral pathways and efficient communication channels between disciplines can enhance continuity and coordination of care.

In addition, workforce limitations and workload distribution require attention to enable effective psychological support. High patient loads, time pressure, and insufficient staffing may restrict nurses’ ability to engage in therapeutic communication. Nurse leaders should therefore advocate for appropriate staffing levels, balanced task allocation, and supportive work conditions that allow adequate time for individualized psychosocial care.

The findings also emphasize the value of involving family members in supporting patients’ emotional well‐being. Nursing leadership should promote family‐centered approaches by encouraging participation in preoperative education, providing emotional reassurance, and, where appropriate, supporting shared decision‐making. Structured guidance for relatives may further reduce patient distress and strengthen coping before surgery.

Finally, mental health promotion should be systematically embedded within institutional protocols for preoperative cardiac care. Psychological support must be regarded as an essential component of comprehensive nursing practice rather than an optional addition. In Kazakhstan, where integration of mental health within clinical services is still evolving, nursing leadership can serve as a key catalyst for practice transformation. Through evidence‐informed protocols, capacity strengthening, and staff empowerment, nurse managers can facilitate the integration of psychological care into routine practice. Overall, these findings underscore the critical contribution of nursing leadership in advancing holistic, patient‐centered care that addresses both physical and emotional dimensions of health.

## 6. Conclusion

The findings indicate that, although nurses demonstrated the use of communication, storytelling, and family involvement as strategies to promote the mental health of preoperative CVD patients, their practice is often constrained by the lack of formal training, standardized protocols, and insufficient institutional support. Persistent challenges in delivering mental health care were also evident, particularly staffing shortages, high nurse‐to‐patient ratios, and restrictive visitation policies, all of which limited the capacity for consistent psychological support. In addition, the results underscored the critical role of family support in enhancing patient well‐being, with nurses actively involving relatives in the care process to strengthen emotional support and coping during the preoperative period.

## Author Contributions

Acquisition of the data: Alpamys Shaldarbek. All authors contributed to the conception and design of the work, the analysis and interpretation of the data, drafting of the manuscript, and critical revision for important intellectual content.

## Funding

The open access publication was supported by Nazarbayev University under the transformative agreement with Wiley.

## Disclosure

All authors provided the final approval of the version to be published and are accountable for all aspects of the work.

## Ethics Statement

This study was approved by the Ethics Committee of Nazarbayev University (NU IREC number RE:2024Nov#01) and by the hospital’s Local Ethics Committee. This research was conducted ethically in accordance with the World Medical Association Declaration of Helsinki.

## Consent

Written informed consent was acquired from all participants prior to the conduct of any study activities. Written informed consent was acquired from all participants prior to the conduct of any study activities. A written consent to publish was obtained.

## Conflicts of Interest

The authors declare no conflicts of interest.

## Data Availability

The data that support the findings of this study are available upon request from the corresponding author. The data are not publicly available due to privacy or ethical restrictions.
